# A phase 1 study of combined guadecitabine and cisplatin in platinum refractory germ cell cancer

**DOI:** 10.1002/cam4.3583

**Published:** 2020-11-01

**Authors:** Costantine Albany, Zeeshan Fazal, Ratnakar Singh, Emmanuel Bikorimana, Nabil Adra, Nasser H. Hanna, Lawrence H. Einhorn, Susan M. Perkins, George E. Sandusky, Brock C. Christensen, Harold Keer, Fang Fang, Kenneth P. Nephew, Michael J. Spinella

**Affiliations:** ^1^ Division of Hematology/Oncology Indiana University Melvin and Bren Simon Comprehensive Cancer Center Indiana University School of Medicine Indianapolis IN USA; ^2^ Department of Comparative Biosciences and the Cancer Center at Illinois University of Illinois at Urbana‐Champaign Urbana IL USA; ^3^ Department of Biostatistics Indiana University School of Medicine Indianapolis IN USA; ^4^ Department of Epidemiology Geisel School of Medicine at Dartmouth Hanover NH USA; ^5^ Astex Pharmaceuticals, Inc Pleasanton CA USA; ^6^ Medical Sciences Program Indiana University School of Medicine Bloomington IN USA

**Keywords:** cisplatin, DNA methylation, DNA methyltransferase, epigenetics, guadecitabine, SGI‐110, testicular cancer

## Abstract

**Purpose:**

Germ cell tumors (GCTs) are cured with therapy based on cisplatin, although a clinically significant number of patients are refractory and die of progressive disease. Based on preclinical studies indicating that refractory testicular GCTs are hypersensitive to hypomethylating agents (HMAs), we conducted a phase I trial combining the next‐generation HMA guadecitabine (SGI‐110) with cisplatin in recurrent, cisplatin‐resistant GCT patients.

**Methods:**

Patients with metastatic GCTs were treated for five consecutive days with guadecitabine followed by cisplatin on day 8, for a 28‐day cycle for up to six cycles. The primary endpoint was safety and toxicity including dose‐limiting toxicity (DLT) and maximum tolerated dose (MTD).

**Results:**

The number of patients enrolled was 14. The majority of patients were heavily pretreated. MTD was determined to be 30 mg/m^2^ guadecitabine followed by 100 mg/m^2^ cisplatin. The major DLTs were neutropenia and thrombocytopenia. Three patients had partial responses by RECIST criteria, two of these patients, including one with primary mediastinal disease, completed the study and qualified as complete responses by serum tumor marker criteria with sustained remissions of 5 and 13 months and survival of 16 and 26 months, respectively. The overall response rate was 23%. Three patients also had stable disease indicating a clinical benefit rate of 46%.

**Conclusions:**

The combination of guadecitabine and cisplatin was tolerable and demonstrated activity in patients with platinum refractory germ cell cancer.

## Graphical abstract

A phase I trial demonstrates tolerable toxicity and clinical activity for combining the hypomethylating agent guadecitabine with cisplatin for testicular cancer patients that have failed prior chemotherapy. This strategy has the potential to be the first targeted treatment for chemotherapy refractory testicular cancer.

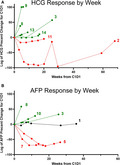

## 1 INTRODUCTION

Germ cell tumors (GCTs) are the most frequent solid cancers of males between the ages of 16 to 39 and have the highest mortality in this age group.^1^ Metastatic GCTs can be cured with conventional therapy based on cisplatin at a rate approaching 80% while common solid cancers are fatal in the metastatic setting.^2^, ^3^ However, 15%–20% of patients are not cured by this treatment. Most patients that relapse after salvage high‐dose platinum die from progressive disease.^4^, ^5^ In addition, mediastinal germ‐cell tumors have especially poor outcomes.^6^, ^7^ To date, targeted therapies for refractory GCTs have not been identified. The young age of GCT patients also results in a relatively large cost in mortality and morbidity.^8^

There are two major types of GCTs, seminomas, and nonseminomas. Nonseminomas consist of embryonal carcinoma (EC), yolk sac tumor, choriocarcinoma, and teratoma.^9^ EC are the putative stem cells of GCTs which are derived from primordial germ cells.^10^ Generally, the DNA of testicular germ cell tumors (TGCTs) is less methylated compared to other cancers, especially seminomas.^11^, ^12^, ^13^ Methylation of RASSF1A, HIC1, MGMT, and CALCA has been shown to correlate with cisplatin resistance.^14^, ^15^, ^16^

For some solid tumor types, there is a correlation between increased DNA methylation and resistance to chemotherapy that can be reversed by hypomethylating agents (HMAs).^17^, ^18^ Resensitization is often accompanied by re‐expression of tumor suppressor genes. A number of preclinical and phase I and phase II clinical studies in ovarian cancer indicate the potential of HMAs as re‐sensitizers in platinum‐resistant disease.^19^, ^20^, ^21^ These include studies with a next‐generation HMA, guadecitabine (SGI‐110), a pro‐drug of decitabine. Guadecitabine has improved pharmacokinetics compared to decitabine.^22^ Guadecitabine has been tested for the treatment of acute myeloid leukemia (AML) and myelodysplastic syndrome (MDS).^22^

It has been reported that EC cell lines from TGCT patients are very sensitive to low doses of the HMAs decitabine and guadecitabine compared to other cancers.^23^, ^24^, ^25^, ^26^, ^27^ Cisplatin‐resistant EC was also sensitive to HMAs and this sensitivity correlated with high levels of DNA methyltransferase 3B (DNMT3B) in the EC cells. Preclinical studies also indicate that HMAs could resensitize cisplatin‐resistant cells to cisplatin when used as a pretreatment strategy.^23^, ^24^, ^25^, ^26^, ^27^

In this current report, we describe the results of a phase I trial combining guadecitabine and cisplatin in a cohort of highly pretreated patients with platinum refractory metastatic GCTs. This protocol mimics preclinical animal studies of testicular cancer. Our study found that combining guadecitabine with cisplatin was tolerable and had clinical activity, providing a rationale for continuing the clinical investigation of this combination for refractory GCTs.

## 2 MATERIALS AND METHODS

### 2.1 Patient population

**Figure 1:**
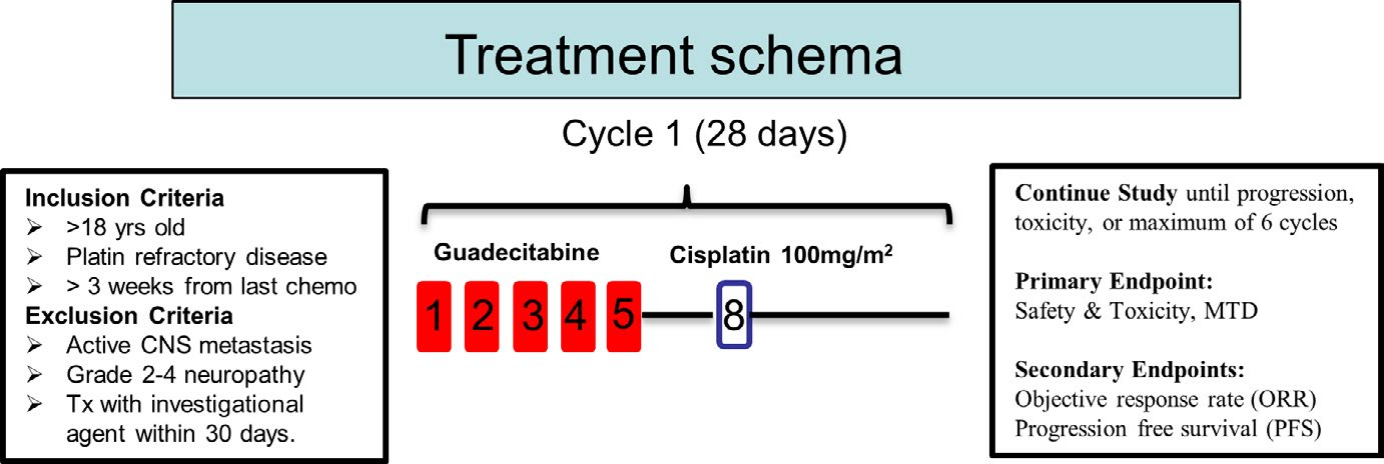
Schema of trial

All patients were registered with the Indiana University Simon Comprehensive Cancer Center Clinical Regulatory Office. Patients 18 years and older who had histologically or serologically confirmed recurrent, metastatic GCTs were eligible. Patients had measurable disease as assessed by Response Evaluation Criteria in Solid Tumors (RECIST) or had increased and clearly rising GCT biomarkers (hCG or AFP).^28^ Patients had acceptable liver and kidney function and an Eastern Cooperative Oncology Group (ECOG) performance status of at least 2.^29^ Patients also needed to be chemotherapy naïve for 3 weeks and have active disease that was platinum refractory. Platinum refractory disease was defined as progression while on cisplatin‐based chemotherapy or within 6 weeks after completing chemotherapy or after high‐dose chemotherapy (HDCT). The criteria for refractory disease in our study were increased to 6 weeks from the typical 4 weeks to increase rate of recruitment.^30^ Exclusion criteria included active, symptomatic central nervous system metastasis, and Grade 2 or greater neuropathy (Figure 1). The protocol was approved by the Institutional Review Board at the Indiana University Simon Comprehensive Cancer Center. Patient‐informed consent was documented. The trial can be found on ClinicalTrials.gov, NCT02429466.

### 2.2 Trial design

The trial was planned as a single‐arm, open‐label phase Ib study of guadecitabine followed by cisplatin (Figure 1). Combination therapy could continue for up to a maximum of six cycles or until progression or intolerable toxicity. The primary objective was to assess the safety and toxicity of guadecitabine plus cisplatin including the dose‐limiting toxicity and the maximum‐tolerated dose. The secondary objective was to assess the efficacy of guadecitabine to resume sensitivity to cisplatin in refractory germ cell tumors. Guadecitabine was given on days 1 to 5 by subcutaneous injection and 100 mg/m^2^ cisplatin was administered by i.v. infusion on day 8 of a 21 or 28‐day cycle. In the dose escalation/de‐escalation design, three patients were anticipated for each dose of guadecitabine with the interval starting at 30 mg/m^2^ (planned escalation to 45 and 60 mg/m^2^ following a modified toxicity probability design^31^) and a 21‐day cycle, based on prior reports for ovarian cancer and hematologic malignancies. Treatment was for six cycles unless there was tumor progression or intolerable toxicity. Prophylactic growth factor support was recommended but not mandatory. Dose‐limited toxicity (DLT) was defined using the Common Terminology Criteria for Adverse Events (CTCAE) v.4.0 and included any drug‐related Grade 3 or Grade 4 non‐hematologic toxicity except Grade 3 or Grade 4 nausea or vomiting that is controllable by anti‐emetics or Grade 3 or 4 diarrhea controlled by optimal therapy.^32^ Grade 4 thrombocytopenia, Grade 3 thrombocytopenia with significant hemorrhage, and febrile neutropenia were considered DLTs. For 45 mg/m^2^ and 60 mg/m^2^ doses, guadecitabine was withheld or dose reduced for Grade 4 myelosuppression or Grade 3 or greater guadecitabine‐related non‐hematologic toxicities until toxicity has resolved to Grade 1 or less or baseline levels. For the dose of 30 mg/m^2^ guadecitabine a dose reduction to 24 mg/m^2^ was allowed. Dose reduction of cisplatin was allowed to 75 mg/m^2^ if there was incidence of Grade 3–4 toxicity. If any dosing was delayed by more than 14 days or more than 2 dose reductions of guadecitabine was indicated because of drug‐related toxicity, patients would be withdrawn from the study. Responders who completed six cycles with tolerable toxicities could elect to be treated with guadecitabine alone every 4 weeks until progressive disease was evident. Follow‐up was every 2 months for the first year off protocol and then every 4 months until death. Patient life expectancy entering the trial was <12 months.

Evaluation of response was determined by RECIST criteria using CT and MRI and also testicular cancer tumor markers (TMs).^33^ All patients with or without measurable disease, were evaluated for TMs (AFP and hCG). The baseline serum AFP needed to be >25 and hCG >10. Complete response was defined as normalization of TM levels (less than the upper limit of normal) that is maintained for at least 21 days in the absence of clinically or radiographically detectable disease. Partial TM response was defined as a decline of at least 50% confirmed by a second measurement more than 3 weeks later.

### 2.3 DNA methylation analysis

For Patient 5, genomic DNA was extracted from formalin‐fixed and paraffin‐embedded (FFPE) samples from C1D1 and C1D8 biopsies and purified using the Qiagen DNeasy assay. Infinium MethylationEPIC beadchip array analysis was performed by the genomics core at the University of Southern California.^34^ DNA was bisulfite treated using the EZ DNA methylation kit (Zymo) and also processed for FFPE restoration by standard protocols. The MethylationEPIC array targets 862,927 CpG sites across the genome. All DNA methylation analysis of MethylationEPIC array was performed in R v.3.6.2. The IDAT intensity files were analyzed in minfi R package.^35^ Raw intensity values were normalized with minfi preprocesser Noob.^35^ The methylation datasets have been deposited in the GEO database under accession number GSE152802.

## 3 RESULTS

### 3.1 Patients

Fourteen patients were enrolled and 13 were treated for at least one cycle (Table 1). One patient withdrew consent after C1D1 and was not evaluated further. The median age was 29 (range, 24–46). All patients had measurable, cisplatin refractory metastatic GCTs. For 12 patients the primary site was the testis and for two patients the primary site was mediastinal. The majority of patients were heavily pretreated with nine patients having prior high‐dose chemotherapy (HDCT) with peripheral blood stem cell transplant and 11 patients progressing after 2nd line therapy and six patients after 3rd line therapy (Table 1). The primary tumors of twelve patients had nonseminoma histology and two had seminoma histology. Of these two patients, one was pure seminoma and the other was of mixed histology of 30% seminoma and 70% nonseminoma. Eleven of the 14 patients were defined as poor‐risk based on International Germ Cell Cancer Collaborative Group (IGCCCG) classification.^36^

**Table 1:** Patient characteristics

Characteristics	Total (*N* = 14)
Age (years)	
Median	29
Range	24–46
Gender, *n* (%)	
Male	13 (92.9)
Female	1 (7.1)
Race, *n* (%)	
White	12 (85.7)
Asian	1 (7.1)
Black or African American	1 (7.1)
Primary site, *n* (%)	
Testis	12 (85.7)
Mediastinal	2 (14.3)
IGCCCG risk at diagnosis, *n* (%)	
Good	2 (14.3)
Intermediate	1 (7.1)
Poor	11 (78.6)
Pathology	
Seminoma	2 (14.2)
Nonseminoma	12 (85.7.6)
Metastatic	14 (100)
Cisplatin refractory	14 (100)
Prior chemotherapy	
1st line (BEP or VIP)	14 (100)
2nd line (HDCT × 2; TICE; TIP)	11 (78.6)
3rd line (taxol/gemcitabine)	6 (42.9)
4th line (etoposide; taxol/gemcitabine)	2 (14.3)

Abbreviations: IGCCCG, International Germ Cell Cancer Collaborative Group; BEP, bleomycin, etoposide, cisplatin; VIP, cisplatin, etoposide, ifosfamide; HDCT, high‐dose chemotherapy; TICE, paclitaxel, ifosfamide, high‐dose carboplatin, etoposide; TIP, paclitaxel, ifosfamide, and cisplatin.

### 3.2 Safety

The primary objective of this study was to assess safety and tolerability and determine MTD. The first cohort of three patients were treated with subcutaneous guadecitabine injected daily days 1–5 at the starting dose of 30 mg/m^2^ followed by cisplatin at 100 mg/m^2^ on day 8 every 21 days. This dose was well tolerated, and the dose of guadecitabine was escalated to 45 mg/m^2^ for cohort 2. However, the first patient of cohort 2 had a DLT and this dose was deemed to be intolerable with Grade 4 neutropenia, Grade 3 febrile neutropenia and Grade 4 thrombocytopenia. Hence, 30 mg/m^2^ was identified as the MTD. It was also noted that patients in cohort 1 needed a delay in treatment of C2D1 of 3 to 5 days for recovery of hematologic counts. Hence the protocol was amended to 30 mg/m^2^ guadecitabine daily for day 1 to 5 followed by 100 mg/m^2^ cisplatin on day 8 every 28 days and DLT criteria were adjusted to accommodate grade 4 thrombocytopenia lasting <7 days. One of the next nine patients on this protocol had an incident of grade 4 thrombocytopenia without bleeding sequelae, resolved to grade 1 upon delaying and reducing the dose of guadecitabine in cycle 2, and continued to complete all six cycles of the protocol. One patient had Grade 3 diarrhea that resolved upon reducing the dose of guadecitabine and cisplatin in cycle 2. In addition, one patient reported Grade 3 dyspnea and one patient reported Grade 3 non‐cardiac chest pain. Due to further preclinical and clinical data suggesting efficacy of low‐dose guadecitabine as a single agent and considering the toxicity profile, three patients were also evaluated at a dose of guadecitabine of 24 mg/m^2^ and cisplatin at 80 mg/m^2^.^23^, ^24^, ^25^, ^26^, ^27^ One patient treated with this protocol had grade 3 anemia that resolved upon delaying and reducing the dose of guadecitabine and cisplatin in cycle 2. In total the most common overall grade 3–4 adverse events were neutropenia (79%), thrombocytopenia (43%), and anemia (36%; Table 2). No treatment‐related deaths were recorded, and no patients discontinued study therapy due to toxicity.

**Table 2:** Summary of all grade 3–4 treatment‐related adverse events

Event	%
Neutropenia	78.6
Thrombocytopenia	42.9
Anemia	35.7
Diarrhea	7.1
Febrile neutropenia	7.1
Hypokalemia	7.1
Hypophosphatemia	7.1

### 3.3 Efficacy

**Figure 2:**
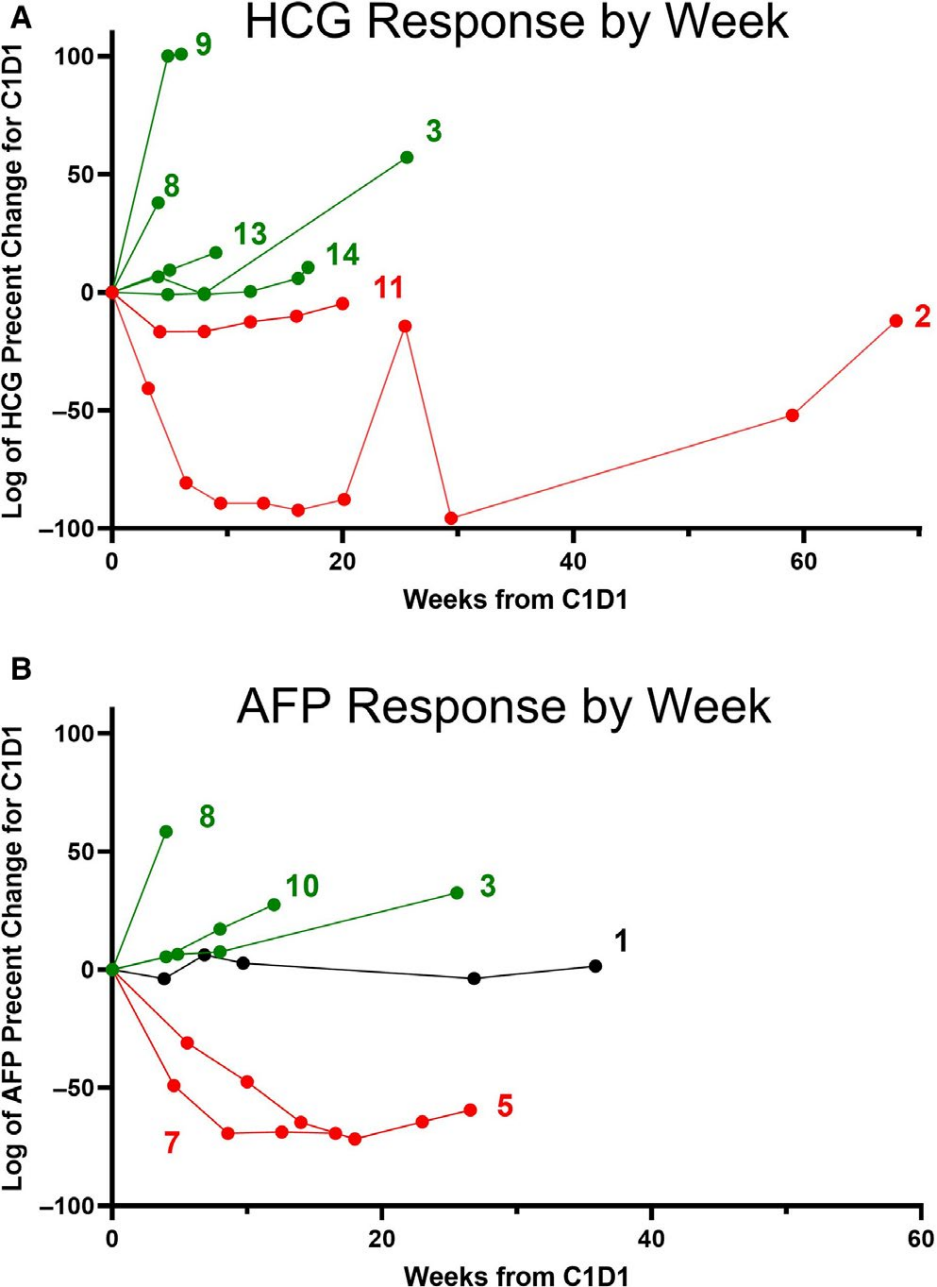
Percent change in testicular germ cell tumor markers as compared with baseline. (A) Log of the percent change in hCG serum levels in individual patients compared to C1D1. Only patients with a C1D1 hCG level >10 are included. Patients with declining levels are in red, patients with increasing levels are in green. (B) Log of the percent change in AFP serum levels in individual patients compared to baseline. Only patients with a baseline AFP level >25 are included. Patients with declining levels are in red, patients with increasing levels are in green. Patient with mixed/unchanged level is in black

One patient of the 14 treated was not evaluable because of withdrawn consent after C1D1. Median follow‐up was 6.9 months (range 0.03 to 26.5) and the median overall survival was 7.8 months (95% CI, 2.7, 12.5). The median progression‐free survival was 1.7 months (95% CI, 0.9, 3.7). The median number of cycles completed was 3. Three patients (Patients 2, 5, and 7) had a partial response as assessed by RECIST.^28^ Two of these patients (Patients 2 and 5) achieved a complete response as assessed by testicular cancer tumor markers AFP or hCG and were still in remission after completing the six cycles (Figure 2). The third partial responder completed five cycles. Patient 2 had metastatic nonseminoma choriocarcinoma treated first line with bleomycin etoposide, cisplatin (BEP) ×4 therapy, second line with HDCT with paclitaxel and ifosfamide followed by high‐dose carboplatin plus etoposide (HDCT TI‐CE) ×3 and third line radiation prior to guadecitabine plus cisplatin. Patient 2 achieved a 13‐month remission and survived for 26 months. Patient 5 had primary mediastinal nonseminomatous germ cell tumor (PMNSGCT) of yolk sac histology and prior first‐line cisplatin, etoposide and ifosfamide (VIP) ×4, second‐line HDCT ×2 (carboplatin), and third‐line gemcitabine plus oxaliplatin before entering the trial. Patient 5 achieved 5‐month progression‐free remission and 16‐month overall survival. In addition, another nonseminoma patient, Patient 7, completed 5 cycles and achieved a partial response assessed by RECIST of 5‐month duration and 8‐month survival (Figure 2). Also three patients (Patients 1, 11, and 14) achieved stable disease as assessed by RECIST. Two of these patients remained on therapy for five cycles (Patients 11 and 14) whereas the third patient with stable disease remained on therapy for three cycles.

**Figure 3:**
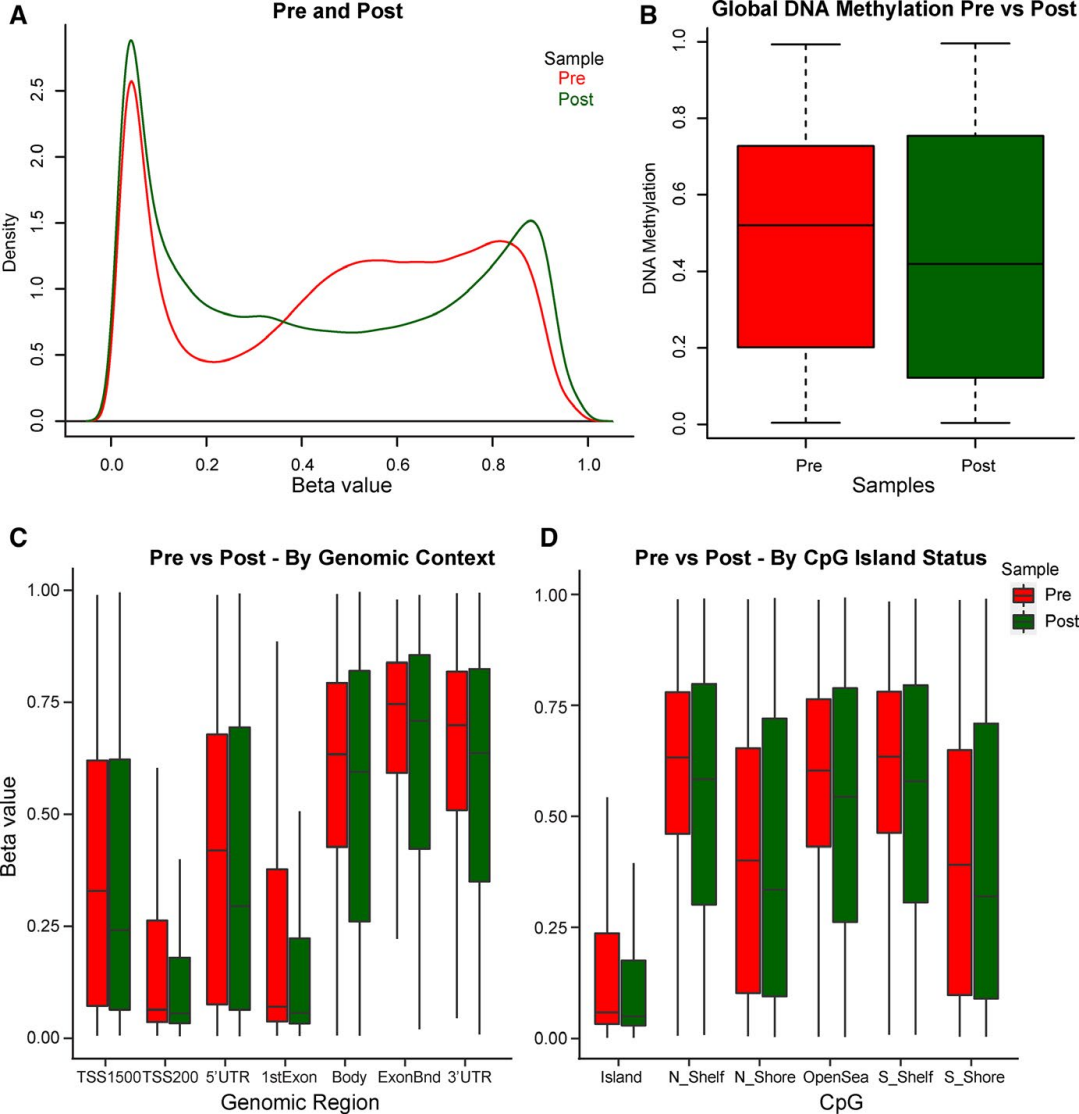
Global DNA methylation levels in complete responder pre and postguadecitabine as assessed by EPIC array. (A) Density of beta values of individual CpG sites for Patient 5 on C1D1, preguadecitabine (red) and on C1D8 after daily C1D1‐C1D5 guadecitabine treatment (green). A substantial decrease in beta values from 0.35 to 0.75 occurred after guadecitabine therapy. (B) The mean of the total beta values pre and postguadecitabine. (C) The mean of the total beta values pre and postguadecitabine, CpG probes stratified by genomic context. (D) The mean of the total beta values pre and postguadecitabine, CpG probes stratified by island status

The serial biopsy of complete responder Patient 5 was analyzed for global CpG methylation using the Infinium MethylationEPIC beadchip array. For Patient 5, there was a decrease in global DNA methylation (Figure 3A,B). The trend in DNA demethylation was consistently observed across sets of CpG probes stratified by CpG island status and gene region (Figure 3C,D).

## 4 DISCUSSION

The concept of re‐sensitization therapy with HMAs is currently being evaluated for solid tumors.^17^, ^18^ There are several reasons to suggest that re‐sensitization therapy may be particularly effective for testicular germ cell tumors (TGCTs). TGCTs appear to have distinct epigenetic profiles, including hypomethylated DNA that has been suggested to correlate with sensitivity to cisplatin.^37^, ^38^ Further the majority of TGCTs can be cured with cisplatin‐based therapy in the metastatic setting, suggesting a potentially large therapeutic benefit in reversing resistance in this setting.^2^ In addition, in preclinical models including xenograft studies, it has been shown that embryonal carcinoma cells, the putative stem cells of nonseminoma TGCTs, are exquisitely sensitive to single‐agent treatment with HMAs compared with common somatic cancer cells, as reviewed.^39^, ^40^, ^41^ TGCT cells are 100‐ to 1000‐fold more sensitive to decitabine and guadecitabine as compared to somatic cancer cells and cisplatin refractory cells could be resensitized to cisplatin.^23^, ^24^, ^25^, ^26^, ^27^ Furthermore, it has been shown in these preclinical studies that low doses of 5‐azacitabine and decitabine‐mediated apoptosis that is dependent on high levels of DNMT3B.^23^, ^24^, ^25^, ^26^, ^27^ Importantly, guadecitabine as a single‐agent dramatically reduced the growth of cisplatin‐resistant EC *in vivo* and resensitized cisplatin‐resistant tumors to cisplatin.^25^ Here we describe a phase I trial assessing the safety and activity of guadecitabine combined with cisplatin in a cohort of highly pretreated, cisplatin‐resistant TGCT patients. It was determined that the MTD was 30 mg/m^2^ guadecitabine on days 1 to 5 and 100 mg/m^2^ cisplatin on day 8 of a 28‐day cycle and that the DLT was neutropenia and thrombocytopenia. This regimen was otherwise well tolerated with the expected hematologic toxicities. We observed clinical activity in this heavily pretreated, platinum‐resistant patient population, including two patients with dramatic responses that are rarely seen in similarly heavily pretreated cisplatin refractory cases. These findings support further testing of DNA hypomethylating re‐sensitization therapy for TGCTs.

HMAs have been assessed in single‐arm trials for ovarian cancer with the combination of decitabine or guadecitabine and carboplatin and a recent study of guadecitabine and irinotecan for metastatic colon cancer. In a phase I trial of 20 patients with recurrent ovarian cancer treated with guadecitabine and carboplatin, partial and stable responses were noted for an ORR of 15% and a CBR or 45%, respectively.^20^ This led to a phase II randomized trial with guadecitabine plus carboplatin compared to physician choice that showed a nonsignificant trend for improvement in overall PFS (16.3 weeks vs. 9.1 weeks).^21^ However, the 6‐month PFS was significantly higher in the guadecitabine plus carboplatin group (37% vs. 11%, *p* = 0.003), validating the potential utility of HMAs as re‐sensitization agents.^21^ A phase I study also demonstrated early promising results for the use of guadecitabine plus irinotecan in metastatic colon cancer.^42^

The MTD of our trial was determined to be 30 mg/m^2^ guadecitabine day 1 through day 5 and 100 mg/m^2^ cisplatin on day 8 of a 28‐day cycle. These findings are similar to phase I studies in ovarian and colon cancer that also utilized 5‐day treatments of guadecitabine and an 8th day treatment with chemotherapeutic agents.^20^, ^42^ The major toxicities we observed neutropenia, thrombocytopenia and anemia were similar to those seen in these studies and were manageable, although the incidence of thrombocytopenia was higher in our study, likely related to the use of cisplatin. As noted in the guadecitabine plus irinotecan study, we recommend mandatory growth factor support for the combination of guadecitabine or other HMAs plus cisplatin in future studies.^42^

Three patients had partial responses by RECIST criteria and two of these patients had complete responses as assessed by serum tumor marker criteria. Both these patients failed and rapidly progressed on multiple prior chemotherapy regimens including HDCT. Major responses are rare in TGCT patients that fail HDCT suggesting the potential utility of guadecitabine for refractory TGCTs. Notably, one of the complete responders had a primary mediastinal nonseminomatous germ cell tumor, which is prone to cisplatin resistance and very difficult to treat in the relapse setting. Interestingly, preclinical studies have found the nonseminoma embryonal carcinoma cells are hypersensitive to HMAs when used as single agents compared to somatic tumors that correlated with very high levels of the DNA methyltransferase, DNMT3B.^23^, ^24^, ^25^, ^26^, ^27^ Hence it is possible that doses substantially lower that the MTD of guadecitabine or other HMAs may be effective in TGCTs as single agents or when combined with other therapies. Our trial was not designed to assess response to guadecitabine alone. All patients who responded had already progressed on platin therapy before the trial, suggesting that the positive response was due to guadecitabine. Further studies are needed to confirm this result and interpretation. While not evaluated in this study, it would be important to assess whether tumor DNMT3B levels correlate with response in future HMA trials in TGCTs with larger numbers of patients enrolled.

There are limitations to our studies. This includes the small sample size and lack of placebo, randomization and blinding as per the phase 1 trial design. Furthermore, we were only able to obtain pre and posttreatment DNA methylation analysis on a single patient. Hence while these preliminary results are promising, follow‐up studies need to be performed to confirm the efficacy of guadecitabine in GCTs. In summary, we report a phase I study evaluating the safety and activity of guadecitabine in combination with cisplatin in refractory GCTs. This combination was tolerable and showed promising activity in the setting a cisplatin‐resistant disease. Our trial paves the way for future studies of HMAs in GCTs, including the recent FDA approved oral decitabine derivative ASTX727.^43^

## CONFLICT OF INTEREST

Dr. Harold Keer is an employee of Astex Pharmaceuticals, Inc.

## AUTHOR CONTRIBUTIONS

Conceptualization, CA, NA, NHH, LHE, HK, KPN, and MJS; Data curation, CA, NA, NHH, SMP, and GES, Formal analysis, CA, ZF, RS, EB, NA, SMP, BCC, FF, KPN, and MJS; Funding acquisition; CA, HK, and MJS; Investigation, CA, ZF, RS, EB, FF, KPN, and MJS; Methodology, ZF, BCC, FF, and KPN; Supervision, CA, NA, NHH, LHE, KPN, and MJS; Visualization, CA, ZF, RS, BCC, FF, KPN, and MJS. Writing ‐ original draft, MJS, Writing ‐ review and editing, NA, NHH, LHE, SMP, BCC, and KPN.

## Data Availability

The methylation datasets have been deposited in the GEO database under accession number GSE152802.
